# Supplementary material for: Missing Heritability in Albinism: Deep Characterization of a Hungarian Albinism Cohort Raises the Possibility of the Digenic Genetic Background of the Disease
Source: Int J Mol Sci. 2024 Jan 20;25(2):1271. doi: 10.3390/ijms25021271 (PMC10817051; doi:10.3390/ijms25021271)
Supplement: Supplementary file 1 [file ijms-25-01271-s001.zip › ijms-2739744-supplementary.pdf]

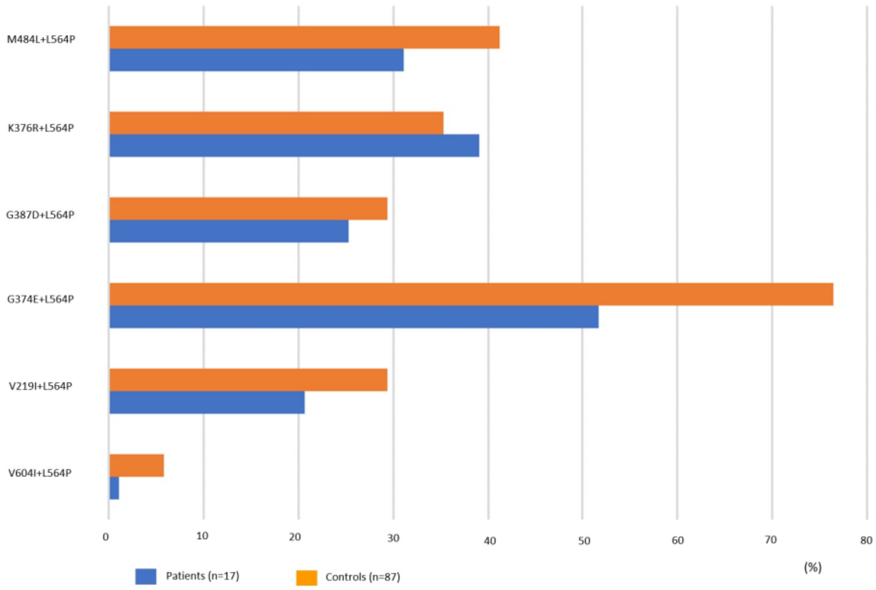

Supplementary Figure 1. The frequencies of the common missense TPC2 variants identified in the Hungarian albinism and control cohorts.

Common TPC2 variants with previously demonstrated gain-of-function effects in combination with the L564P variant, M484L, K376R and G387D, were detected with similar frequencies among patients and controls. No significant difference was observed between patients and controls for the allele frequencies of any of the detected frequent missense variants of the *TPCN2* gene in this Hungarian cohort. In addition to carrying the L564P variant, all investigated individuals (n = 93) carried at least one or more frequent missense variant (s) of the *TPCN2* gene: on average, three missense variants were present in the patients and two in the controls. The detected common missense *TPCN2* variants are not linked.

**Clinical symptoms**	**Patient No. 16**
Skin colour	Hypopigmented, no tanning ability
Hair colour	Light blondish
Eye colour	Blue
Eye abnormality	Foveal hypoplasia

Supplementary Table 1.

The clinical characteristics of patient No. 16.
